# ^99m^Tc-HYNIC-(tricine/EDDA)-FROP peptide for MCF-7 breast tumor targeting and imaging

**DOI:** 10.1186/s12929-018-0420-x

**Published:** 2018-02-19

**Authors:** Sajjad Ahmadpour, Zohreh Noaparast, Seyed Mohammad Abedi, Seyed Jalal Hosseinimehr

**Affiliations:** 1 Department of Radiopharmacy, Faculty of Pharmacy, Mazandaran University of Medical Sciences, Sari, Iran; 2 Student Research Committee, Mazandaran University of Medical Sciences, Sari, Iran; 3 Department of Radiology, Faculty of Medicine, Mazandaran University of Medical Sciences, Sari, Iran

**Keywords:** Breast cancer, MCF-7, Tumor targeting, Imaging, FROP, Radiopharmaceutical

## Abstract

**Background:**

Breast cancer is the most common malignancy among women in the world. Development of novel tumor-specific radiopharmaceuticals for early breast tumor diagnosis is highly desirable. In this study we developed ^99m^Tc-HYNIC-(tricine/EDDA)-Lys-FROP peptide with the ability of specific binding to MCF-7 breast tumor.

**Methods:**

The FROP-1 peptide was conjugated with the bifunctional chelator hydrazinonicotinamide (HYNIC) and labeled with ^99m^Tc using tricine/EDDA co-ligand. The cellular specific binding of ^99m^Tc-HYNIC-FROP was evaluated on different cell lines as well as with blocking experiment on MCF-7 (human breast adenocarcinoma). The tumor targeting and imaging of this labeled peptide were performed on MCF-7 tumor bearing mice.

**Results:**

Radiochemical purity for ^99m^Tc-HYNIC-(tricine/EDDA)-FROP was 99% which was determined with ITLC method. This radiolabeled peptide showed high stability in normal saline and serum about 98% which was monitored with HPLC method. In saturation binding experiments, the binding constant (K_d_) to MCF-7 cells was determined to be 158 nM. Biodistribution results revealed that the ^99m^Tc-HYNIC-FROP was mainly exerted from urinary route. The maximum tumor uptake was found after 30 min post injection (p.i.); however maximum tumor/muscle ratio was seen at 15 min p.i. The tumor uptake of this labeled peptide was specific and blocked by co-injection of excess FROP. According to the planar gamma imaging result, tumor was clearly visible due to the tumor uptake of ^99m^Tc-HYNIC-(tricine/EDDA)-FROP in mouse after 15 min p.i.

**Conclusions:**

The ^99m^Tc-HYNIC-(tricine/EDDA)-FROP is considered a promising probe with high specific binding to MCF-7 breast cancer cells.

## Background

Breast cancer is the second most common malignancy with high risk of metastasis and death among women in the world [1]. The 5-year survival rate for women diagnosed with metastatic breast cancer is only about 27.4% [2]. In order to overcome mortality and morbidity in breast cancer, diagnosis of this cancer in the early stage is necessary. The early detection can dramatically improve the survival rate of breast cancer patients [3]. So far traditional imaging methods, such as x-ray computed tomography (CT), ultrasound and magnetic resonance imaging (MRI), have been used for breast cancer diagnosis [4]. Mammography is a commonly used screening method for breast cancer. This method can be problematic due to low sensitivity especially in the case of dense breasts [5–7]. Therefore, high sensitive methods for detecting of breast cancer at its earliest presymptomatic stages are necessary. Single-photon emission computed tomography (SPECT) or positron emission tomography (PET) imaging techniques represent an appropriate tool with high sensitivity for breast cancer imaging. In recent years developing a peptide-based radiopharmaceuticals due to certain favorable properties for the detection of breast cancer are increased. Previously technetium-99m labeled alpha-M2 [8], H6F [9], TP1623 [10], PI [11] and EC peptide [12] are used as a molecular imaging agent for breast cancer.

A 12-amino-acid FROP-1 peptide as tumor cell binding peptide was identified through phage display system by Sabine Zitzmann research group [13]. FROP-1 peptide consists of Glu^1^-As^2^-Tyr^3^-Glu^4^-Leu^5^-Met^6^-Asp^7^-Leu^8^-Leu^9^-Ala^10^-Tyr^11^-Leu^12^ amino acids that significantly has ability binding to the various type of the cancer cell lines, especially thyroid cancer cells FRO82–2 and MCF-7 breast cancer cells. Previously this research group labeled FROP-1 peptide with iodide radioisotopes (^131^I and ^125^I) [13] and also ^111^In radionuclide [14] for in vitro and in vivo evaluation of FROP-1 binding to MCF-7 cell line.

^99m^Tc is one of the most commonly used radionuclides in the clinic. The popularity of this radionuclide is due to its appropriate half-life and excellent radiochemical properties that make it suitable for peptide-based nuclear imaging. The use of a HYNIC as a bifunctional chelating ligand for the labeling of a various biomolecules, including antibodies and peptides has received considerable interest [15, 16].

In radiolabeling method, the nature and location of a chelating moiety within a peptide molecule can influence on its receptor-binding. To avoid possible interference of the radionuclide with the binding region of peptides, the spacer is incorporated between the binding site and the chelating moiety. For completing the coordination sphere of the technetium core in ^99m^Tc-HYNIC the presence of the co-ligand is required. The stability, lipophilicity, clearance property, and protein binding potency of the radiolabeled compounds are affected by the type of a co-ligand [17]. In the labeling process, co-ligands tricine and EDDA could be used separately or together via the exchange labeling method.

In Zitzmann and Mire research, since the labeling method and pharmacokinetics characterization of FROP-1 radiopeptide derivatives have problems of slow binding capacity and insufficient in vivo accumulation, and furthermore with respect to the effect of the type of chelator and co-ligand on radiopharmaceutical’s pharmacokinetic [18, 19] and tumor targeting in animal model [20], we decided to modify FROP-1 peptide through practicing HYNIC chelator. This modification is leading to some advantages including a well-defined chemistry, ability for post conjugation labeling, the ability of storing for later radiolabeling process and production of a stable complex with ^99m^Tc radionuclide [21] which has high popularity in nuclear medicine due to the suitable photonic characterization. Therefore in this study, we radiolabeled FROP-1 peptide through practicing HYNIC as a chelating moiety (at the C-terminus) and Lys residue as a spacer in order to develop a ^99m^Tc-HYNIC-(tricine-EDDA)-Lys-FROP for the targeting of the MCF-7 breast cancer cells.

## Methods

### Materials

The 12-amino acid FROP peptide with HYNIC conjugation at C-terminus (EDYELMDLLAYLK-(HYNIC)) was purchased from Prote-Genix (France). Tricine (*N-[tris-(*hydroxymethyl) methyl] glycine), ethylene diamino diacetic acid (EDDA), trifluoroacetic acid (TFA) and tin (II)-chloride dihydrate, were obtained from Sigma (St. Louis, MO, USA). Acetonitrile (HPLC grade), sodium succinate dihydrate and methyl ethyl ketone (MEK) were purchased from Merck (Darmstadt, Germany). Solutions were prepared by standard procedures and using high quality water. Sodium pertechnetate (^99m^TcO_4_^−^Na) was obtained from commercial ^99^Mo/^99m^Tc generator (Pars Isotope, Tehran, Iran). The radiochemical purity was performed by instant thin layer chromatography (ITLC) with different mobile phases using a Lablogic mini scan TLC scanner (Sheffield, UK) and Laura image analysis software. A NaI (Tl) gamma detector (Delshid, Tehran, Iran) was used for measurement of radioactivity. Reversed phase-HPLC was performed on a Knauer HPLC systems (Knauer HPLC, Berlin, Germany) using a Lablogic radioactivity gamma detector with a Eurospher 100–5 C-18, 4.6 × 250 mm (Knauer, Berlin, Germany) column with a pre-column (Knauer HPLC, Berlin, Germany). MCF-7 (breast cancer), SKOV-3 (ovarian cancer), PC-3 (prostate cancer), A-549 (non-small cell lung cancer), T47D (breast cancer) and HFFF2 (normal human fibroblast) cell lines were obtained from the Iranian Pasteur Institute and the National Center of Genetic and Biological Reserves of Iran. Dulbecco’s DMEM high glucose culture, fetal bovine serum (FBS), penicillin–streptomycin and trypsin solution were purchased from Gibco (UK) and Biosera (UK). All cell lines were grown in Dulbecco’s DMEM high glucose culture media with 10% fetal bovine serum (FBS) in tissue culture flasks in humidified air containing 5% CO_2_ at 37 °C until a sufficient number of cells were available. Confluent cultures were harvested by employing a trypsin solution (Biosera, UK).

### Radiolabeling

For radiolabeling, HYNIC-FROP peptide (10 μg) was incubated with 50 μL tricine solution (10 mg tricine in 50 μL succinate buffer with pH 6.5) and 100 μL succinate buffer. This mixture reaction was shaken and then 25 μL of tin (II) solution (2 mg/1 mL in nitrogen-purged 0.1 M HCL) was added. After adding of the 5 mCi (185 MBq) fresh ^99m^TcO_4_^−^Na and then 150 μL EDDA solution (5 mg EDDA) the mixture reaction heated for 20 min at 95 °C.

The radiochemical purity was determined by instant thin layer chromatography (ITLC) in different mobile phases such as methyl ethyl ketone (MEK), acetonitrile/water (50/50) and citrate solvent (0.1 M, pH 5.2) using a Lablogic mini scan TLC scanner (Sheffield, UK) and Laura image analysis software. In MEK, free ^99m^TcO_4_^−^ migrated with the solvent front, while radiolabeled peptide remained at the application point. In the acetonitrile/water system, the colloids remained at the application point, while radiolabeled peptide migrated in front of solvent. The amount of labeled co-ligand was determined by citrate solvent, that free ^99m^TcO_4_ Na and labeled co-ligand migrate in front of solvent while both RHT and labeled peptide (^99m^Tc-FROP) remind at the origin. A NaI (Tl) gamma detector (Delshid, Tehran, Iran) was used to for measurement of radioactivity.

Reversed phase-HPLC was performed on a Knauer HPLC systems (Knauer HPLC, Berlin, Germany) using a Lablogic radioactivity gamma detector with a Eurospher 100–5 C-18, 4.6 × 250 mm (Knauer, Berlin, Germany) column with a pre-column. (Knauer HPLC, Berlin, Germany) HPLC was run using a gradient of 0.1% TFA in H_2_O (solvent A) and 0.1% TFA in CH_3_CN (solvent B) at a flow rate of 1 mL/min. A gradient with solvents A and B was run as follows: 0 min, 10% B; 0–10 min, 10–30% B; 10–20 min, 30–80% B; and 20–25 min, 80–100% B, for a total time of 30 min.

### Stability in normal saline and serum

The stability of ^99m^Tc-HYNIC-FROP in normal saline was evaluated using ITLC at various times (2, 4, 6 and 24 h) at room temperature. For serum stability, ^99m^Tc-HYNIC-FROP (30 μL) was incubated with freshly human plasma serum (90 μL) in triplicate at 37 °C. At 1 h of incubation, the plasma proteins were precipitated with a mixture of acetonitrile/ethanol (1:1 *v*/v) and centrifugation. The supernatant layer was filtered through a 0.22 μm filter and then the fraction was analyzed by RP-HPLC. Partition coefficient of ^99m^Tc-HYNIC-FROP was determined via method that previously was described [22].

### In vitro binding experiments

The cellular uptake studies of ^99m^Tc-HYNIC-(tricine/EDDA)-FROP were performed on MCF-7, A-549 SKOV-3, PC-3, T47D and HFFF2 cell lines. The cells (5 × 10^5^) were seeded in 12-well plates overnight and incubated with ^99m^Tc-HYNIC-FROP (40 nM) at 37 °C for 1 h. Incubation of the samples was terminated by removing medium and washing with cold serum-free medium. Thereafter, the cells were detached by trypsin and the radioactivity in the cell suspension was quantified.

For specific binding experiment, the MCF-7 cells were saturated with 500-fold excess of unlabeled peptide prior adding of the ^99m^Tc-HYNIC-FROP to cells for 30 min.

### Internalization study

For internalization experiment, MCF-7 cells (1 × 10^6^ cells) were seeded on the single plates and incubated with ^99m^Tc-HYNIC-FROP (40 nM) at 4 °C. After 1 h incubation, medium was removed and cells were washed with ice-cold serum free medium. Then 1 mL complete medium was added to each dish and cells were further incubated for 0.5, 1, 2, 6 and 24 h at 37 °C. After incubation, medium was removed from cellular plate and then was washed once with cold serum free medium. Then supernatant was collected, and for removing radioactivity bound to the surface of the cells, the acid wash with urea buffer (pH 2.5) was used. The internalized radiolabeled peptide was recovered by solubilizing the cells with 1 M NaOH at 37 °C for 5 min, and the surface-bound (acid-wash) and internalized (acid-resistant) radioactivity were measured with the gamma counter.

### Affinity calculation

The cell-binding affinity of ^99m^Tc-HYNIC-FROP was assessed with saturation method. For determining of dissociation rate constant (K_d_) and total number of binding sites (Bmax), ^99m^Tc-HYNIC-FROP was prepared in high radiochemical purity. MCF-7 cells were seeded into 24-well plates and incubated overnight. Then cells were incubated with various concentrations of radiolabeled peptide (30–250 nM) for 30 min. One dish was added blocking solution containing of 500-fold excess of highest concentration of ^99m^Tc-HYNIC-FROP. The cells were then washed with cold serum free medium to remove free radioactivity. The cells with bound radioactivity detached with trypsin and collected and radioactivity was measured. The (K_d_) and (Bmax) value were calculated using Graphpad prism software based on specific binding of ^99m^Tc-HYNIC-FROP on MCF-7 cells.

### Biodistribution in MCF-7 tumor-bearing nude mice

The all animal studies were approved by Research and Ethical Committees of Mazandaran University of Medical Sciences. For xenografting, female C57 nu/nu mice were injected subcutaneously on the right upper back with a suspension of 1 × 10^7^ MCF-7 cells and Matrigel® (BD, USA) and the tumors were allowed to grow for about 7–8 weeks. Estradiol valerate was injected subcutaneously into nude mice for 1 week. After tumor size reached in 1 cm^3^, this tumor was removed from mouse in aseptic condition and was mechanically squelched with a mortar and with adding complete medium. Squelch of tumor tissue was centrifuged at 1500 rpm for 7 min at 4 °C. The upper medium was removed from tube and then complete medium and estradiol were added to cells pellet. Cell suspension (200 μL) was injected to nude mouse implantation site and it was observed at regular intervals for tumor formation and progression. Xenografts were allowed to develop during 2 months and the average tumor size was 1.01 ± 0.24 g. The in vivo biodistribution of ^99m^Tc-HYNIC-(tricine/EDDA)-Lys-FROP was performed on tumor bearing mice at 15 and 30 min post-injection. The mice (four mice in each time group) were injected with 100 μL of HYNIC-(tricine/EDDA)-Lys-FROP (1 μg) via a lateral tail vein. Mice were sacrificed with injection of a lethal dose of ketamine/xylazine after 15 and 30 min. The blood was collected from the heart puncture and the mice were dissected. Major organs (tumor, blood, heart, lung, liver, kidney, spleen, salivary gland, stomach, intestine, muscle and bone) were placed into pre weighed gamma counter tubes and radioactivity in each organ and tissue was measured using a gamma counter and expressed as a percent injected dose (% ID/g).

To confirm that the radiolabeled peptide was taken up specifically by MCF-7 tumor in vivo, the biodistribution of ^99m^Tc-HYNIC-FROP was performed with co-injection of a 500-fold (0.5 mg) excess non-radiolabelled FROP-1 and radiolabelled peptide in mice (*N* = 3 mice in this group). The unlabeled peptide was injected at 30 min before the injection of radiolabeled peptide.

### Tumor imaging

For gamma camera imaging, MCF-7 tumor bearing mouse was intravenously injected through the tail with ^99m^Tc-HYNIC-FROP. After 15 min p.i. the mouse immediately was anesthetized using ketamine/xylazine and whole-body dynamic imaging was conducted. The imaging was performed at the department of radiology at Mazandaran Hospital by a dual-head e.cam gamma camera (Siemens Medical Systems, Inc.), equipped with a low-energy, high-resolution collimator. The evaluation of the image was performed using an e-soft (Siemens Medical Systems).

### Statistical analysis

All statistical analyses were performed using Excel software (2011, Microsoft Office). The in vitro experiments were performed three-times and the final values were presented as mean ± standard deviation (SD). The t-test was used for comparison of groups and *p < 0.05* was considered as statistical significance level between groups. The binding data were analyzed with nonlinear regression using Prism 5 software (Version 5.04, 2010, USA).

## Results

### Radiochemical assessments

**Fig. 1:**
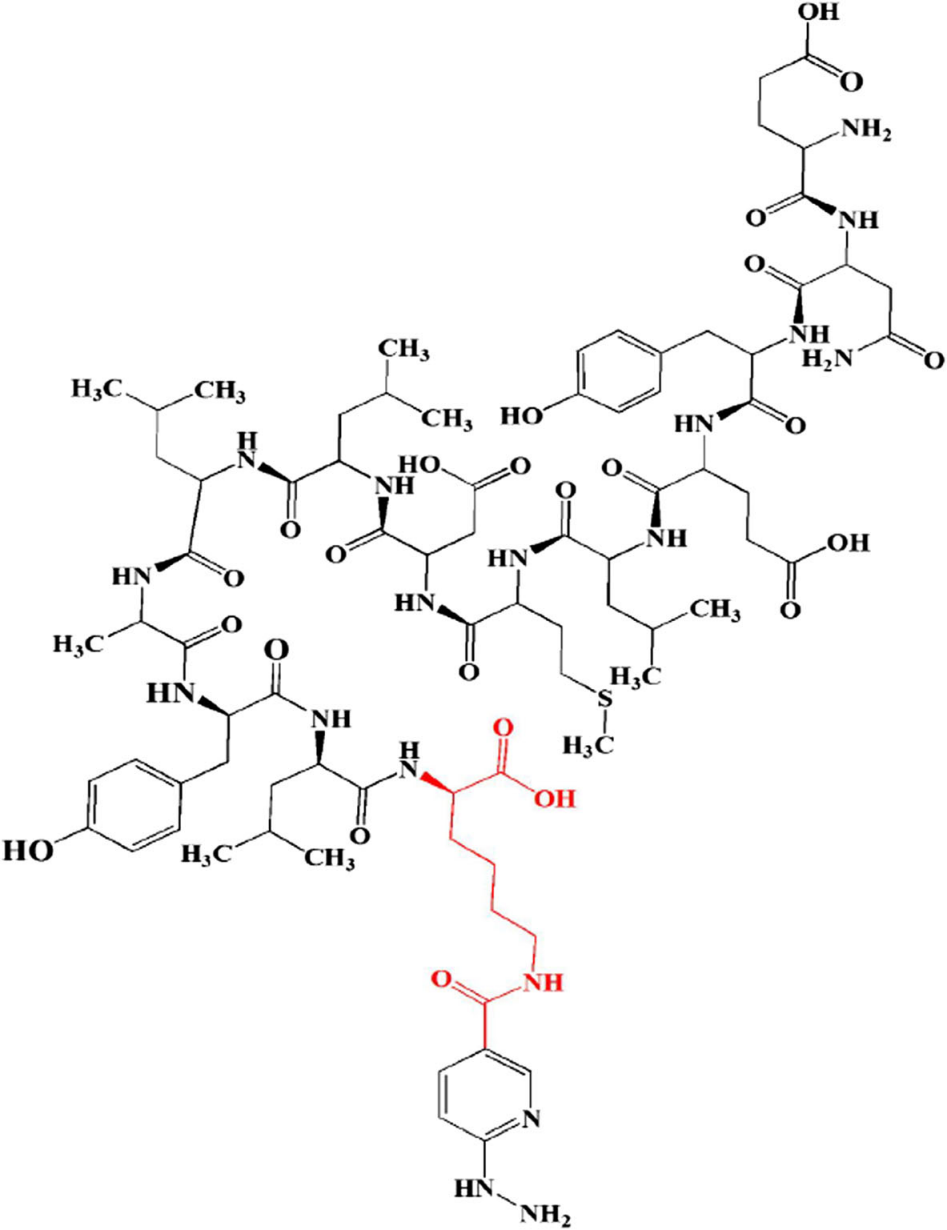
The chemical structure of the HYNIC-K-FROP peptide

**Fig. 2:**
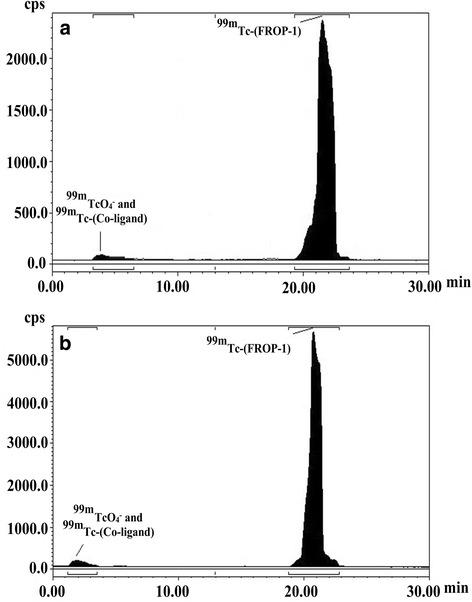
Radio high-performance liquid chromatography (HPLC) analysis of ^99m^Tc- HYNIC-(tricine/EDDA)-FROP peptide at 1 h after labeling (**a**) and incubated in human serum at 1 h after labeling (**b**). The retention time of peptide was from 20 to 22 min and for mixture of ^99m^TCO_4_^−^ and ^99m^Tc-co-ligand was 3–5 min

HYNIC-Lys-FROP (Fig. 1) was labeled efficiently with ^99m^Tc by a ligand exchange method with a radiochemical purity of 99.6% which was determined with ITLC method. Radio-HPLC analysis of ^99m^Tc HYNIC-(tricine/EDDA)-Lys-FROP (Fig. 2a) showed a single radioactive peak at 20–22 min. The high stability of the ^99m^Tc-HYNIC-Lys-FROP was observed to be 99% up to 6 h in normal saline which was determined with ITLC method (Table 1). The HPLC analysis of the human plasma revealed that ^99m^Tc-HYNIC-FROP was stable during incubation at 37 °C with human plasma serum after 1 h (Fig. 2b). The lipophilicity of the radiolabeled peptides was evaluated according to their proportional distribution between octanol and PBS. The partition coefficient for the ^99m^Tc-FROP was log *p* = − 2.03 ± 0.03 (Table 1).

**Table 1:** In Vitro Stability of ^99m^Tc-HYNIC-(tricine/EDDA)-FROP in succinate buffer and normal saline at room temperature *(n = 3)*

%RCP^a^	%RCP in succinate buffer (peptide reaction mixture)				%RCP in normal saline				Log p^b^
^99m^Tc-FROP	2 h	4 h	6 h	24 h	2 h	4 h	6 h	24 h	−2.03 ± 0.03
	99.6 ± 0.2	99.5 ± 0.4	99.1 ± 0.9	96.8 ± 2.4	99.5 ± 0.4	99.1 ± 0.9	99 ± 0.8	94.6 ± 2.7	

^a^RCP = Radiochemical purity that was determined with TLC method, ^b^Partition coefficient between n-octanol and PBS

### Cellular uptake

**Fig. 3:**
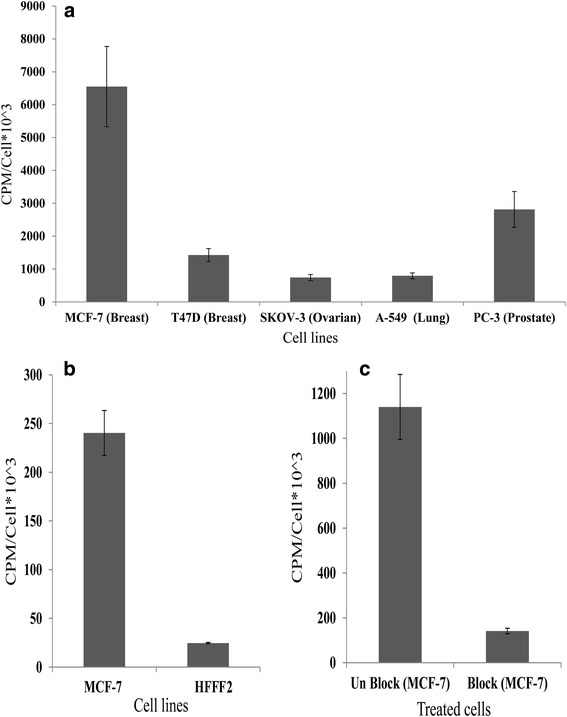
**a** The ability binding of ^99m^Tc- HYNIC-(tricine/EDDA)-FROP peptide on MCF-7 cells and comparison of this binding with binding to the SKOV-3 (ovarian cancer), A-549 (non-small cell lung cancer), T47D (breast cancer) and PC-3 (prostate cancer) cell lines and **b** HFFF2 (normal human fibroblast) cell line. The data are shown as the means ± SD. Data indicates significant difference (*p* values< 0.0001) in uptake of radiolabeled peptide between different cancer and normal cell lines. **c** In vitro specific binding of ^99m^Tc-HYNIC-(tricine/EDDA)-FROP to MCF-7 cells. Pre-saturation was done by blocking unlabeled FROP (500-fold excess) peptide after 0.5 h incubation at 37 °C

For cellular binding experiment, different human cancerous cell lines and normal human fibroblast cell line were used to determine the specific binding of ^99m^Tc-HYNIC-Lys-FROP. The percentage of cell-associated radioactivity of ^99m^Tc-HYNIC-FROP was higher for MCF-7 cells in comparison to the other cancer cell lines (Fig. 3a) and HFFF2 (Fig. 3b). The MCF-7/SKOV-3, MCF-7/A-549, MCF-7/T47D and MCF-7/PC-3 ratios for ^99m^Tc-FROP uptakes were 8.8, 8.2, 4.5 and 2.3 respectively. Also the binding of ^99m^Tc-HYNIC-FROP peptide to the non-tumor cell lines were obtained very low compared to MCF-7 cell line, to the point that MCF-7/HFFF2 ratio was found 9.7. The results of in vitro cell binding competitive assay that were obtained by addition of 500-fold excess of unlabeled peptide (FROP) as a competitor, showed significant reduction of ^99m^Tc-HYNIC-FROP binding to the MCF-7 cells which was clearly demonstrated that this binding is through FROP-1 peptide (Fig. 3c).

**Fig. 4:**
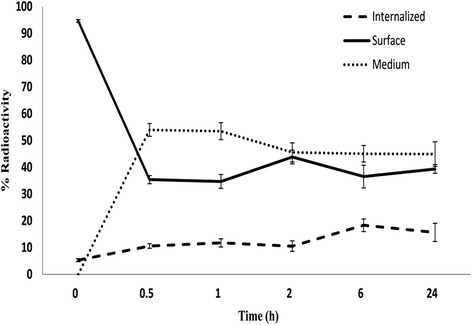
Internalization of the ^99m^Tc-HYNIC-(tricine/EDDA)-FROP peptide by MCF-7 cells at different times after incubation at 37 °C: total binding represents uptake on the surface membrane plus that inside of the cell; internalization represents the activity inside of the cell only

The in vitro internalization experiment was performed to determine the rate and fraction of internalization of ^99m^Tc-HYNIC-FROP in MCF-7 human breast cancer cells. The percentage of internalized radiolabeled peptide was 10.6 ± 0.8% of the total radioactivity in the cells after 30 min in the human breast cancer cells while after 6 h it was 18.3 ± 2.3%. The results of in-vitro time dependency internalization of ^99m^Tc-HYNIC-FROP are shown in (Fig. 4).

### Binding affinity

**Fig. 5:**
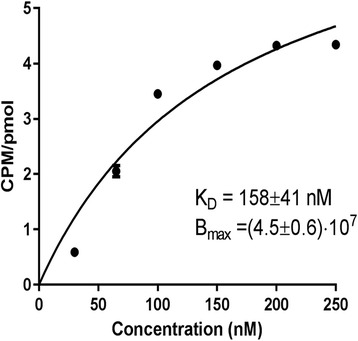
Saturation binding assay curves of ^99m^Tc-HYNIC-(tricine/EDDA)-FROP peptide upon incubation with MCF-7 cells

The receptor-binding affinity (K_d_) and maximum number of binding sites (B_max_) of ^99m^Tc-HYNIC-(tricine-EDDA)-Lys-FROP were determined on MCF-7 cells. The K_d_ and B_max_ values for radiolabeled peptide were calculated to be 158 ± 41 nM and 4.5 ± 0.6 × 10^7^ CPM/pMol respectively (Fig. 5).

### In vivo animal biodistribution

The results of the biodistribution studies demonstrated that the ^99m^Tc-HYNIC-FROP rapidly cleared from the blood circulation due to the mainly renal exertion. The uptake values of ^99m^Tc-HYNIC-FROP in MCF-7 tumors were 0.25 ± 0.02 and 0.38 ± 0.21% ID/g at 15 min and 30 min p.i., respectively. This tumor accumulation was specific and confirmed with reduced tumor uptake (0.09 ± 0.02 ID/g) for block mice group at 15 min p.i. (Table 2). The kidneys showed the highest amount of radioactivity, 2.2 %ID/g at 15 min, but decreased to 1.73 ID/g% after 30 min. The highest tumor-to-muscle ratio was observed at 15 min p.i. and equal to 3.03 ± 0.12. The results of the biodistribution studies and tumor-to-tissue ratios of ^99m^Tc-HYNIC-FROP are summarized in Table 2.

**Table 2:** Biodistribution and tumor-to-tissue ratios of ^99m^Tc-HYNIC-(tricine/EDDA)-FROP in MCF-7 female tumor-bearing nude at 15 and 30 min p.i. Data expressed as % ID/g organ

^99m^Tc-HYNIC-FROP			
	%ID/g		
	15 min	30 min	Block-15 min
Tissue			
Blood	0.48 ± 0.09	0.35 ± 0.10	0.69 ± 0.16
Heart	0.49 ± 0.08	0.26 ± 0.11	0.45 ± 0.03
Lung	0.45 ± 0.08	0.41 ± 0.26	0.60 ± 0.04
S.T^a^	0.25 ± 0.03	0.32 ± 0.22	0.25 ± 0.01
Liver	0.20 ± 0.02	0.14 ± 0.00	0.26 ± 0.04
Spleen	0.15 ± 0.01	0.28 ± 0.11	0.23 ± 0.07
Kidney	2.22 ± 0.16	1.73 ± 0.15	2.97 ± 0.23
Stomach	0.21 ± 0.01	0.33 ± 0.12	0.26 ± 0.01
Muscle	0.08 ± 0.01	0.21 ± 0.16	0.15 ± 0.01
Bone	0.18 ± 0.02	0.24 ± 0.12	0.22 ± 00
Intestine	0.34 ± 0.09	0.79 ± 0.25	0.64 ± 0.09
Tumor	0.25 ± 0.02	0.38 ± 0.21	0.09 ± 0.02
Tumor/Tissue			
Muscle	3.03 ± 0.12	1.94 ± 0.46	0.60 ± 0.12
Blood	0.53 ± 0.15	1.09 ± 0.47	0.14 ± 0.06
Spleen	1.61 ± 0.22	1.56 ± 1.04	0.42 ± 0.14
Bone	1.36 ± 0.28	1.57 ± 0.48	0.40 ± 0.09
Liver	1.24 ± 0.28	2.62 ± 1.38	0.36 ± 0.13

^a^Salivary Gland and Thyroid

### Tumor imaging

**Fig. 6:**
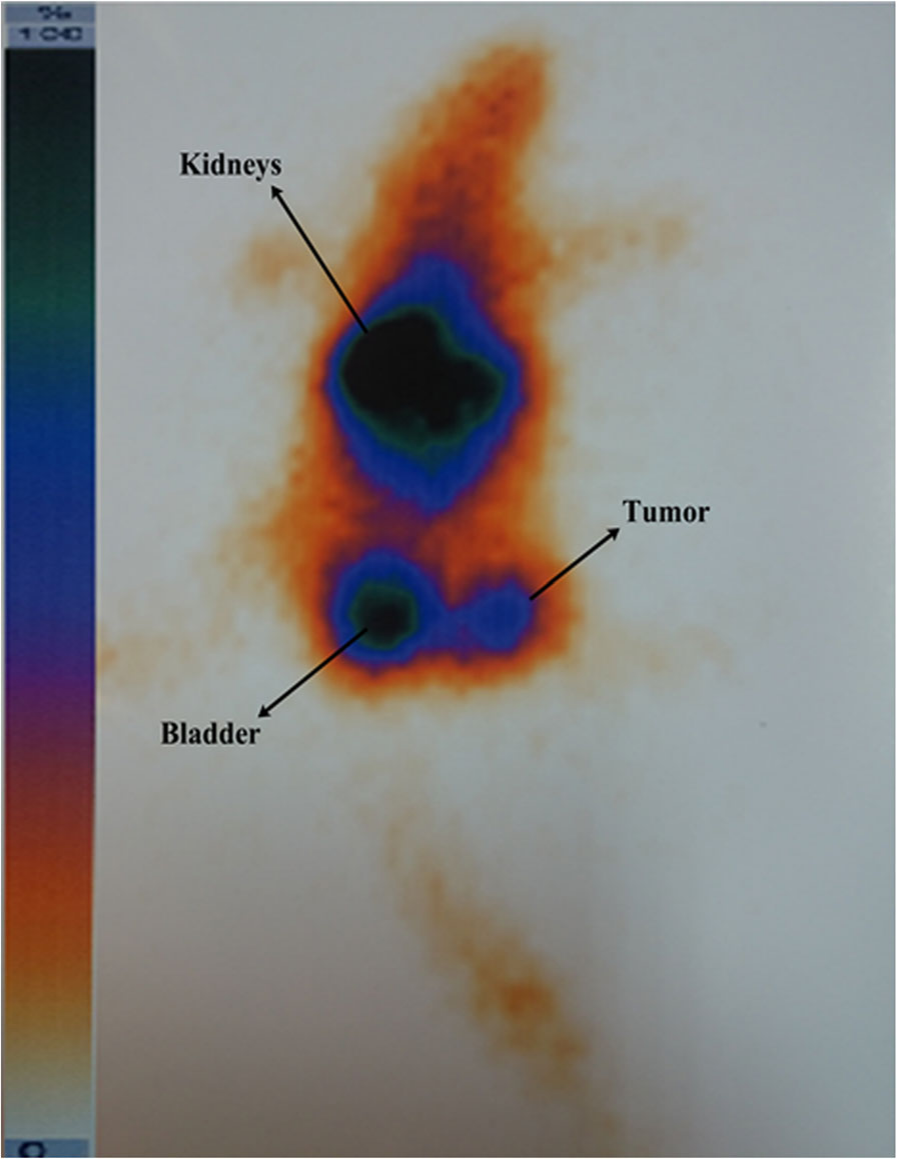
Imaging of MCF-7 breast cancer xenograft nude mouse using ^99m^Tc-HYNIC-(tricine/EDDA)-FROP peptide; planar γ-camera images were acquired at 15 min after injection

Tumor imaging using ^99m^Tc-HYNIC-FROP was performed in MCF-7 female tumor-bearing nude mice. The planer gamma imaging was acquired at 15 min after the administration of ^99m^Tc-HYNIC-FROP. According to the scintigraphy result, tumor was clearly visualized by SPECT imaging at 15 min p.i. (Fig. 6). However, kidneys and bladder uptake is high due to the fast clearance of radiolabeled peptide from blood circulation.

## Discussion

Breast cancer is one of the most common causes of cancer with high mortality among women in the world [2]. It is necessary to mention that diagnosis of breast cancer at early stage leads to significantly improve the efficacy of therapy and an overall reduction of breast cancer mortality. Nuclear imaging techniques, namely, PET and SPECT enable to detect tumors at early stage or metastasis [23]. However various radiopharmaceuticals with high selectivity have been used for molecular imaging in breast cancer [24, 25], but radiolabeled peptide has advantages such as small size, rapid distribution, clearance and high priority target for cancer. Radiolabeled peptide which was used for the targeting of MCF-7 cells is highly interesting. MCF-7 is a commonly used breast cancer cell line that isolated from the pleural effusion of a woman with metastatic disease and is used ubiquitously in research for breast cancer cell experiments [26]. MCF-7 is estrogen receptor (ER) positive cell line with moderate EGFR-expressing and HER-2 negative [27]. MCF-7 parental cells also maintain the expression of epithelial markers, such as E-cadherin, β-catenin and cytokeratin 18 (CK18) and other specific molecular markers of natural epithelial layers, such as claudins and zona occuldens protein 1 (ZO-1), among other proteins that constitute the intercellular junctions. Furthermore, MCF-7 are mesenchymal markers negative, these markers are including vimentin and smooth muscle actin (SMA) [28, 29]. FROP-1 peptide was identified by phage display technology by Sabine Zitzmann research group in 2007 [13]. This research group demonstrated the specific binding of iodinated derivative (^131^I and ^125^I radionuclide) of the FROP-1 peptide for MCF-7 breast tumor cells both in vitro and in vivo models. FROP-1 peptide is not a derivative of cholecystokinin-B or its family which was confirmed by the Basic Linear Alignment and Search Tool against protein databases. Though so far no attempt was made to identify a receptor for the FROP-1 peptide, further more study seems to be needed to find out the ability of targeting characteristics of this molecule specially targeting of the epithelial markers [13]. Although this agent exhibited a specific binding in cellular experiment, its in vivo tumor uptake was limited due to poor stability and pharmacokinetics. This limitation was due to the fact that the iodination of tyrosine occurred in radiolabeling reaction. The mixture of different by-products not only was difficult to purification as a single product but also the existence of this impurity affected on binding of the peptide to the receptor. However, several methods were conducted for improvement the stability and affinity of the FROP-1 molecule such as coupling of FROP-1 peptide to chelator [14] and its conjugating with ethylene glycol (PEG) oligomer [30]. In comparison to the FROP-1 peptide without chelator, coupling of a FROP-1 peptide to the chelator 1, 4, 7, 10-tetraazacyclododecane-1, 4, 7, 10-tetraacetic acid (DOTA) led to enhancing cellular uptake kinetics and increasing in vivo stability. High stability of FROP-DOTA is attributed to the different secondary structure between FROP-DOTA and free FROP-1 peptide in solution which was conformed by circular dichroism (CD) spectra [14]. However the binding kinetic was found to be too slow and its fast excretion by urinary system forestalled on tumor accumulation. To solve this problem, PEGylation of ^111^In-DOTA-FROP was proposed by Walter Mire. The conjugation of FROP-DOTA with PEG oligomer increased a time dependent tumor uptake, but unfortunately the binding kinetics of the PEGylated ^111^In-DOTA-FROP was even slower than that of ^111^In-DOTA-FROP [30]. With respect to the effect of changing chelator on pharmacokinetic of radiopharmaceuticals [18, 19, 31], in this research study we developed a new radiolabeled FROP peptide derivative using the HYNIC as a chelating moiety at C-terminus and EDDA-tricine as co-ligand to form a stable complex with ^99m^Tc by ligand exchange method. Mainspring choosing of C-terminus for labeling was that the attachment of the chelator to the C-terminal end stabilize the peptide against enzymatic degradation and induce structural constrains that resemble those in the phage-bound structure [14]. FROP-1 peptide was labeled with ^99m^Tc at high radiochemical purity (≥99.5%). It is necessary to mention that the type of buffer which was used for labeling reaction affected the radiochemical purity. The maximum radiochemical purity was found for succinate buffer (pH 6.5) without any further purification. In comparison to the other buffer it is most likely that succinate buffer assemble good situation for facilitating the exchange radiolabeling reaction. Also ^99m^Tc-HYNIC-FROP showed a good in vitro stability in normal saline and human serum and after 1 h incubation with freshly human serum that the major radioactivity was still associated with ^99m^Tc-FROP. This in vitro and in vivo stability for FROP peptide attributed to the attachment of HYNIC chelator like that was seen for DOTA-FROP. To validate the ability of ^99m^Tc-HYNIC-Lys-FROP as a ligand to detect MCF-7 breast cancer cells, we evaluated the cellular uptake of the peptide in various types of a cancer cell lines and normal human fibroblast cell line. Data analysis has indicated that ^99m^Tc-HYNIC-FROP has higher uptake in MCF-7 cells in comparison especially to the normal cells and other cancerous cell lines. Similar to ^111^In-DOTA-FROP the cell binding characteristics of ^99m^Tc-HYNIC-FROP is not affected by the addition of a chelating moiety and chelation with ^99m^Tc. However in comparison to the ^131^I–FROP-1 and DOTA-FROP, the modification of our proposed molecule is leading to not only the improvement of binding capacity and binding kinetics but also the in vivo tumor accumulation. This binding was specific and significantly blocked in the presence of cold peptide as a competitor. The internalization experiment showed that the fast internalization was occurred after 30 min the exposure of MCF-7 cell with radiolabeled peptide. However, the amount of internalization for ^99m^Tc-HYNIC-FROP was greater than both ^125^I–FROP-1and ^111^In-DOTA-FROP at 30 min by a factor of approximately 2. ^99m^Tc-HYNIC-FROP revealed a high rate of internalization between 4 and 6 h after incubation perhaps due to the both slow internalization kinetics and the attachment of the HYNIC chelator leading to high stability of the FROP-1 peptide against the peptidases of the MCF-7 cell surface [32]. This delayed internalization was previously seen for ^111^In-DOTA-FROP in Mire research but not for ^131^I–FROP-1 [14].^99m^Tc-HYNIC-FROP exhibited a higher affinity in the nanomolar range (158 nM) for MCF-7 cell than ^125^I–FROP-1 that was in micromolar range (8 μM). The dissociation constant in the nanomolar range for ^99m^Tc-HYNIC-FROP demonstrates the high specific affinity to the MCF-7 cells. Therefore, conjugation of the HYNIC moiety to FROP-1 peptide increases the affinity by a factor of approximately 78. However the binding affinity of ^111^In-DOTA-FROP to FRO82–2 cells was reported 494 nM [14]. The in vivo results showed the radiolabeled peptide exhibited a rapid clearance from the blood. The excretion pathway was mainly through renal system which possibly was attributed to the nature of peptide sequence and the amphiphilic character of the FROP-1 peptide with a log *p* = − 2.03. The same excretion pathway was seen for ^131^I–FROP-1 [13]. In comparison with ^131^I–FROP-1, both DOTA-FROP and HYNIC-FROP showed a faster blood clearance. This rapid blood clearance is caused by the hydrophilic DOTA and HYNIC conjugates that have high polarity. A low uptake of radioactivity was seen in the stomach and thyroid at all times, indicating a high in vivo stability for ^99m^Tc core in ^99m^Tc-HYNIC-FROP peptide. The tumor uptake of ^99m^Tc-HYNIC-FROP peptide was found to be 0.25 ± 0.02% ID/g after 15 min that this tumor uptake relatively good (in comparison the kidneys uptake). However, this tumor uptake was specific and significantly blocked in the presence of the cold peptide (FROP-1) as a competitor in block mouse group. In most organs (except the excretion organs) the excess of unlabeled peptide did not lead to a change in radiolabeled peptide accumulation. Both radiolabeled HYNIC-FROP and DOTA-FROP (For MCF-7 cell line) peptides showed similar kinetics for tumor accumulation, as the steady decrease of radioactivity in tumor was detected after 15 min p.i. [14]. Unfortunately, the fast wash out of ^99m^Tc-HYNIC-FROP peptide from tumor caused low tumor uptake compared to lung and heart which is a limiting factor for this radiolabeled peptide. The maximum tumor/muscle ratio was obtained after 15 min p.i. of ^99m^Tc-HYNICFROP the same time achieved by DOTA-FROP; while this ratio was found for ^131^I–FROP-1 after 135 min. Although previously reported PEGylation of DOTA-FROP increased the tumor/muscle ratio to 4.1 in female Balb/c nu/nu mice bearing FRO82–2 tumor after 24 h p.i. This time is not suitable for imaging goal especially regarding to the short half-life radionuclide. Despite fast washing out from tumor, ^99m^Tc-HYNIC-Lys-FROP has a good ability for tumor visualization after 15 min p.i. This ability can be attributed to the both high Bmax value and high affinity for MCF-7 cells. Also the fast internalization of ^99m^Tc-HYNICFROP in tumor organ immediately after the injection can help to this aim. Though attachment of the HYNIC chelator to FROP-1 peptide improved the both binding capacity and binding kinetics, the further studies are recommended for improvement the in vivo differentiation of tumor and other normal organs. The other co-ligands, spacers and chelators can be practiced for labeling of FROP peptide with ^99m^Tc to achieve better in vivo tumor targeting and imaging.

## Conclusion

In this study, FROP-1 peptide was radiolabeled with ^99m^Tc via HYNIC chelator and formed a stable complex. ^99m^Tc-labeled peptide exhibited highly specific binding on MCF-7 cells that were confirmed in vitro and in vivo. The high affinity of FROP-1 peptide on MCF-7 cells is retained due to attachment of HYNIC chelator and labeling with ^99m^Tc. Biodistribution in mice exhibited a rapid clearance from the blood and excretion pathway was mainly through renal system. Moreover ^99m^Tc-HYNIC-(tricine/EDDA)-FROP accumulated in MCF-7 tumor rapidly and it was observed a high tumor-non-targets ratios. The result of this study is promising for imaging and diagnosis of MCF-7 breast cancer with ^99m^Tc-HYNIC-(tricine/EDDA)-FROP peptide, however future experiments are needed for improvement of in vivo tumor targeting.
